# Promoting Digital Proficiency and Health Literacy in Middle-aged and Older Adults Through Mobile Devices With the Workshops for Online Technological Inclusion (OITO) Project: Experimental Study

**DOI:** 10.2196/41873

**Published:** 2023-02-08

**Authors:** Anna Quialheiro, André Miranda, Miguel Garcia Jr, Adriana Camargo de Carvalho, Patrício Costa, Margarida Correia-Neves, Nadine Correia Santos

**Affiliations:** 1 Life and Health Sciences Research Institute School of Medicine University of Minho Braga Portugal; 2 ICVS/3B’s PT Government Associate Laboratory Braga Portugal; 3 Department of Physiotherapy North Polytechnic Institute of Health, CESPU Famalicão Portugal; 4 Associação Centro de Medicina P5 School of Medicine University of Minho Braga Portugal; 5 Office of Infrastructure and Operations in Information Technology University of Southern of Santa Catarina Tubarão Brazil; 6 Regenerative Medicine Center Utrecht University Medical Centre Utrecht Utrecht Netherlands

**Keywords:** digital proficiency, health literacy, older adults, mobile devices

## Abstract

**Background:**

Digital inclusion and literacy facilitate access to health information and can contribute to self-care behaviors and informed decision-making. However, digital literacy is not an innate skill, but rather requires knowledge acquisition.

**Objective:**

The present study aimed to develop, conduct, and measure the impact, on digital and health literacy, of a digital inclusion program aimed at community dwellers.

**Methods:**

The program targeted the recruitment of people aged 55 and older that owned mobile devices with an internet connection in 3 cities in northern Portugal (Paredes de Coura, Guimarães, and Barcelos). The program was titled the Workshops for Online Technological Inclusion (OITO) project and, in each city, was promoted by the coordinator of municipal projects and organized as an in-person 8-workshop program, using mobile devices, smartphones, or tablets. A quasi-experimental design was used with a nonrandomized allocation of participants in each set of 8 workshops. Sociodemographic, health status, and mobile use information were collected at baseline. Digital and health literacy were measured via the Mobile Device Proficiency Questionnaire and the Health Literacy Scale questionnaires, respectively, at baseline (T1), program completion (T2), and a 1-month follow-up (T3). A self-reported measure of autonomy was evaluated at T1 and T2 using a visual scale.

**Results:**

Most participants were women with primary schooling (up to 4 years) aged between 65 and 74 years and retired. The intervention had an 81% (97/120) recruitment rate, 53% (43/81) adherence, and 94% (67/71) satisfaction rate, with 81 participants completing the entire 8-workshop program. Most participants had owned their mobile device for more than one year (64/81, 79%), were frequent daily users (70/81, 86%), and had received their mobile device from someone else (33/64, 52%). Over 80% (71/81) of the participants who completed the intervention used Android smartphones. At baseline, participants had low baseline scores in digital literacy, but medium-high baseline scores in health literacy. They showed significant improvement in digital literacy at T2 and T3 compared to T1, but without a significant difference between T2 and T3, regardless of sex, age, or schooling. A significant improvement in self-reported autonomy was observed at T3 compared with baseline. Regarding health literacy, no significant differences were found at T2 or T3 compared to the baseline.

**Conclusions:**

The feasibility indicators showed that the OITO project methodology had a substantial rate of recruitment and satisfaction. Program participants had significant improvement in digital literacy after 8 workshops and maintained their score 1 month after completing the intervention. There was no significant change in health literacy during the project period.

## Introduction

The World Health Organization (WHO) lists combating social isolation in older adults as a major target of worldwide health policy [1], and the United Nations Agenda 2030 indicates that society must strive to guarantee “inclusive and equitable quality education and promote lifelong learning opportunities for all” [2]. In the present context of an unprecedented demographic shift in our society, along with the desire and need for older adults to age in place, it is crucial to establish innovative strategies that work to bolster health promotion, paired with literacy programs designed to reduce the loss of independence in the older years. Increased life expectancy is usually a hallmark of a country’s health, but this is meaningless if not accompanied by good quality of life [3]. Because digital technology is increasingly present in everyday tasks, digital literacy is becoming an unavoidable necessity for a good quality of life [4].

Older adults constitute the most excluded population in our information and communication digital society [5-8]. However, the popularization of smartphones with internet access has grown substantially, with these devices being able to replace computers for many of their functions. Thus, individuals who were previously digitally excluded now have, within reach, equipment that allows more traditional forms of communication (ie, telephone calls) combined with online capabilities [6]. The smartphone has opened opportunities for digital inclusion, reinforcing social ties and access to information in various domains [9].

Digital inclusion facilitates access to health information and can contribute to self-care behaviors and informed decision-making about health [10]. Internet use is also associated with functional independence, increased cognitive function, and better mental health [11-13]. The concept of digital literacy is the result of the increasing permeation of digital media in our society and their use in everyday life. There is an increasing demand for using, searching for, and finding digital information, assessing its reliability, and actively selecting and applying it. These are not innate skills. On the contrary, they require knowledge acquisition and suggest an inherent need for the availability of digital literacy programs. The need is particularly relevant for older adults, with a fundamental societal gap becoming apparent—the vulnerability, or societal divide, experienced by older citizens in keeping up with digital phenomena [14]. In addition to digital literacy, older adults face barriers that make it difficult to use digital technologies. A study tested 2 mobile health (mHealth) apps and showed 28 severe usability issues, with most related to the motivational and cognitive barriers of older adults [15]. Promoting digital inclusion for older adults requires addressing (1) the increased need for innovative digital literacy and inclusion programs with proper measures of effectiveness and outcomes and (2) the need for group or social interventions as a central element in any strategy [16-18]. Still, studies have shown that the engagement of older adults in the use of coaching programs for health care is greater than that of young people [19,20], and a qualitative study showed that using social media apps positively impacted health through their ability to keep older adults cognitively engaged, improve health communication, and increase social connectedness [21].

In Portugal, data from the Communication Observatory (Obercom) indicates that 32.9% of the Portuguese population aged 55 to 64 years used the internet in 2013, rising to 65.3% in 2020, but in the next age group (≥65 years) only 11.8% used the internet in 2013 and 39% in 2020 [22,23]. According to the Survey on the Use of Information and Communication Technologies by Families carried out by the Portuguese National Institute of Statistics in 2020, the northern Portuguese region had the lowest rate of internet use (74.3%). The available data do not differentiate internet usage rates by age group or show broadband internet access [24], but data from Obercom show that in 2019, for the first time, the use of smartphones by the general Portuguese population was greater than the use of computers in searching for information on the internet [25].

In this context, this study aimed to develop, conduct, and measure the impact, on digital and health literacy, of an in-person 8-workshop guided digital inclusion project, the OITO (the Portuguese abbreviation for Oficinas de Inclusão Tecnológica Online, “Workshops for Online Technological Inclusion”) project, for using mobile devices with an internet connection; the project was aimed at community dwellers aged 55 years or older in 3 northern Portuguese cities.

## Methods

### Study Design, Participants, and Data Collection

The OITO project had a quasi-experimental design with a convenience sample of participants in each set of 8 workshops. Participants were included if they (1) were aged 55 years or older, (2) dwelled in the community, (3) owned a mobile device with internet connection or capability, and (4) were enrolled in 1 of 3 ongoing, municipal-wide projects in cities in northern Portugal: Paredes de Coura (the Couração project), Guimarães (the Vida Feliz project), or Barcelos (the Municipal Library project). An invitation to participate in the program was made by an OITO project partner via telephone using the number registered by the participant for enrollment and participation in the municipal project. The required sample size of 90 participants was determined based on a 1-tailed sample test, an effect size of *d*=0.35 (between low, 0.2, and medium, 0.5), a type I error (α) of .05, and statistical power (1-β error probability) of .95. This calculation used G-Power (version 3.1.9.6, Heinrich Heine University) [26]. A total of 98 potential participants were recruited. Attendance at the first OITO project workshop confirmed enrollment, and on the same day, the participants were asked to confirm their ownership and use of a mobile device (regardless of perceived aptitude or frequency of use) and to sign the informed consent form. The aim of the study was clearly explained to the participants. Four individuals declined to participate in the program due to health problems or lack of interest in the content, and 7 declined due to transportation difficulties in reaching the location of the workshops. A final sample of 87 participants was thus included in the study (60 in Paredes de Coura, 9 in Guimarães, and 18 in Barcelos).

Data on sociodemographics, lifestyle habits, health status, type of device, frequency of device use, and the autonomy of the participants were collected at baseline (T1). Autonomy data were also collected at T1 and immediately after completing the intervention (T2). Digital and health literacy data were collected at T1, T2, and at 1 month after the intervention (T3). The 16-question Mobile Device Proficiency Questionnaire (MDPQ-16) [8,27] was used to measure digital literacy. No instruments were found to assess digital literacy that had been validated for the Portuguese population; however, the MDPQ-16 constitutes a digital proficiency assessment instrument with generalizable and inclusive components and, therefore, it is adequate for measuring digital literacy in older adults [8]. The MDPQ-16 was adapted by a professional native Portuguese-speaking translator. The instrument’s score ranges from 8 to 40, and it is divided into eight domains: (1) mobile device basics, (2) communication, (3) data and file storage, (4) internet, (5) calendar, (6) entertainment, (7) privacy, and (8) troubleshooting and software management. Each question has the same number of possible answers, with an assigned score of 1 to 5 points. The total score was obtained by adding the mean values in each of the 8 domains. The short-form, 12-question Health Literacy Scale (HLS-12) [28] was used to measure health literacy. The final score was obtained by summing the value of each question and varied between 0 and 4, for a maximum total of 48 points. Both outcomes were analyzed as a continuous number (*c*).

The questionnaires were completed by the participants after they were clearly explained. Help was provided to participants who had any difficulty.

### Intervention

The OITO project intervention’s methodology was adapted from the Memory Workshop (in Portuguese: Oficina da Lembrança), a cognitive stimulation program that uses a computer connected to the internet [29,30]. In the OITO project, the intervention was carried out with the participants’ mobile device, smartphone, or tablet, according to their choice. The program comprised 8 workshops, with meetings held in person on 2 alternate days of the week at a location hosted by the specific project partner. Each workshop lasted approximately 1.5 hours, subdivided into 45 minutes of digital activity, 10 minutes of physical activity, 30 minutes of digital activity, and 5 minutes of exchanging experiences in a “conversation circle.”

The digital activity encompassed a group of apps that were chosen based on the level of stimulation and learning skills: (1) a definition or configuration app and an email app, to train the users in touch and manual dexterity for mobile use (with the definition or configuration apps) and touch and device handling for mobile use as motor skills stimulation (with the email app); (2) WhatsApp and a camera app, for training in exploratory capacity for multiple forms of communication (ie, text, image, voice, and video); (3) a gallery app, Google Photos, YouTube, and Google Fit for training in interactive tasks, such as sharing pictures, video, and information; and (4) WhatsApp for messaging for e-learning and orientation about autonomous activities to improve autonomy by cooperation.

The workshops were led by an experienced trainer in teaching older adults how to use technology. One or two monitors further aided in assisting the OITO project participants throughout the workshops. Each workshop had the following steps: (1) the trainer explained the purpose of the app covered in the session, (2) the trainer demonstrated how to use the app with detailed, step-by-step instructions, and (3) the participants practiced using the app. If any difficulty arose, the trainer and the monitors resolved it individually or collectively, as needed. The trainer and monitors did not touch the participants’ mobile devices, but rather indicated what the participants should do. This allowed the participants to actively and independently use their devices, instead of just observing or relying on someone doing it for them.

The brief physical activity moment consisted of standing exercises to stimulate circulation and balance training. The conversation circle consisted of participants, the trainer, and the monitors reporting their perception of the day’s activities. All workshops were hosted by the same team of researchers with no deviation from the program between locations. Each workshop had no more than 10 participants, with a ratio of 2 instructors to 5 participants. All participants received a project manual with all the steps necessary to use the apps covered throughout the workshops [31]. The OITO project took place between May 2021 and January 2022.

### Measures

Digital literacy was analyzed as the primary outcome, using the MDPQ-16. The secondary outcome was health literacy, measured by the HLS-12. The adjusted self-reported variables were sex (male or female), age (determined by birth year and categorized into groups: 55-64, 65-74, and ≥75 years), and educational level (according to the Portuguese system of education). The educational level was transformed into categories (years of formal schooling): primary education (1-4 years); basic education (5-9 years); secondary education (10-12 years); and higher education (more than 12 years).

Participants self-reported marital status (single, married, divorced, or widowed), household composition, and job status (retired, employed, or unemployed). For household composition, participants were asked to report the number of people currently living at their home (single occupancy, married or cohabiting with no dependent children, married or cohabiting with dependent children, single parent, or other multi-person).

Physical activity level, chronic pain, incidence of hypertension and diabetes, and smoking and alcohol habits were self-reported as dummy variables (yes or no). Study participants also reported the average duration of physical activity sessions and how many days per week they exercised. The average session time was multiplied by days per week to estimate the total minutes of exercise per week. According to the WHO recommendations for moderate-intensity aerobic physical activity in older adults (aged 65 years or older), this score was categorized into >150 minutes, <150 minutes, and 0 minutes [32].

Because the digital inclusion program targeted the recruitment of people with mobile devices, and we confirmed that the participants were, in some form, already users of their mobile devices, health problems that could be barriers to the use of the device, such as reduced vision, attention, memory, or fine motor skills, were not evaluated.

Participants were asked about mobile device–related characteristics, including who purchased the device (self or other), how often they used the device (frequent users were defined as those who used it every day or multiple times a day, with all others being infrequent users), and how long they had owned the device (more or less than 1 year). In addition, information about the type (smartphone or tablet) and operating system (Android or Apple iOS) of the mobile device was recorded.

Self-reported autonomy was assessed with the following item: “Circle the value corresponding to how autonomous you feel when using a mobile phone with a touchscreen/tablet, with 1 being very little autonomous and 10 being completely autonomous.” The options were 1 to 10 on a visual scale that included numbers and colors (as a gradient from red to green).

The OITO project intervention was evaluated by 3 feasibility indicators: recruitment, adherence, and workshop satisfaction. The recruitment rate was calculated as the sum of all recruited individuals and divided by the number of vacancies (10 per workshop). The intervention adherence rate was determined by the number of participants who attended all workshops and the total number of workshop participants. Participants’ satisfaction with the proposed intervention was measured using a 5-point Likert scale (from very dissatisfied to very satisfied) at the end of the 8 workshops.

### Statistical Analysis

The Shapiro-Wilk test, which we applied for the scale variables, indicated significant deviation from a normal distribution. A transversal analysis was performed to determine the characteristics of the participants according to their digital and health literacy baseline data. Central tendency and dispersion measures were used to characterize the sample.

The psychometric properties of the MDPQ-16 were measured through an exploratory factorial analysis (EFA) using the principal axis factoring extraction method and calculating the Cronbach α and McDonald ω as reliability measures. The Kaiser-Meyer-Olkin (KMO) test ensured sampling adequacy (KMO=0.862). The Bartlett test of sphericity for the EFA model (*χ*^2^_28_=308; *P*<.001) revealed proper correlation structures for factor analysis. The Cronbach α (α=.880) and McDonald ω (ω=0.883) revealed excellent internal consistency.

An independent 2-tailed *t* test and 1-way ANOVA (or the Welch version when the assumption of equal variances was not met) were used to compare 2 or more groups. The nonparametric alternatives, the Mann-Whitney test and Kruskal-Wallis test, were used for the baseline data. An inferential analysis to compare the variables at T1, T2, and T3 was performed using a paired-sample 2-tailed *t* test and a repeated-measures ANOVA was used to analyze differences between the 3 time points. The Cohen *d* was used for effect size calculation for the *t* test. The association between digital and health literacy was analyzed using linear regression. A crude analysis and an analysis adjusted by sex, age, and education was performed. A longitudinal analysis model with generalized estimating equations (GEEs) was used to estimate the effect of time on digital literacy. The GEE analysis extends the generalized linear model that accounts for the within-subject correlation across repeated measures and considers time as a variable. The database reading changes from “wide” to “long,” permitting the analysis of within-subject missing data.

*P* values <.05 with 95% CIs were considered significant. The software used for descriptive analyses was Jamovi (version 1.6.23; The Jamovi Project), and the software used for inferential and compared analysis was StataMP (version 14.0; StataCorp).

### Ethical Procedures

The study was approved by the regional Ethics Committee for Health and the Life and Health Sciences Ethics Committee of the University of Minho (CEICVS 101/2021). The participants received detailed information about the study’s procedures and objectives and agreed to participate by signing the voluntary informed consent form. This study used the Transparent Reporting of Evaluations with Nonrandomized Designs (TREND) checklist.

## Results

### Sociodemographic and Mobile Use Characteristics

Of the 87 individuals enrolled in the OITO project, 81 completed the intervention (ie, they attended the 8 workshops that the program was composed of). Of these, 66.7% (54/81) were women, 70.4% (57/81) were married, 59.3% (48/81) were between 65 and 74 years old, 44.4% (36/81) had up to 4 years of education, and 91.4% (74/81) were retired. The majority (34/81, 42%) lived only with a spouse or partner. Approximately 70% (57/81) of the participants were from Paredes de Coura, 22% (18/81) from Barcelos, and 7% (6/81) from Guimarães. Regarding device characteristics, 88.9% (72/81) of the participants were smartphone users, and 87.7% (71/81) had a device with the Android operating system. A family member bought the mobile device for, or gave it to, most participants (33/64, 51.6%); 79% (64/81) had owned their device for more than 1 year, and 86.4% (70/81) were frequent users (Table 1).

We assessed the association of baseline health and digital literacy according to sociodemographics, mobile device characteristics, and health characteristics of the OITO project participants (Table 1). The scores for the literacy questionnaires showed that participants had low baseline scores in digital literacy and medium-high baseline scores in health literacy. We calculated the proportion of participants with a baseline score on the questionnaires of more than half the maximum, because the questionnaires did not have a cutoff point. If a participant’s score was more than half the maximum, they were considered to have higher literacy. For digital literacy, only 19% (17/79) of participants had a score equal to or greater than 24 points, with a range from 8 to 40 points. For health literacy, 85.3% (70/80) of participants scored higher than 24 points, with a range from 0 to 48 points. Significant differences were observed across municipalities for both types of literacy at baseline. Participants who had purchased their own device, had a smartphone (versus a tablet), used it frequently, or had owned the device for more than a year had better digital literacy scores. Participants with a higher education level had better digital and health literacy scores than those with less education. Finally, those who practiced regular physical activity had better health literacy scores.

Seventy-one percent (58/81) of the participants reported being physically active or exercising at least once a week, and 28.4% (23/81) described themselves as physically inactive. Among the physically active participants, 35.4% (28/79) exercised more than 150 minutes per week, and 35.4% (28/79) exercised less than 150 minutes per week. Among noncommunicable chronic diseases, 56.8% (46/81) had hypertension, 13.9% (11/79) diabetes, and 45% (36/70) chronic joint pain. As for lifestyle habits, 1.2% (1/79) were smokers and 5.1% (4/79) consumed alcohol outside main meals.

**Table 1:** Baseline sociodemographics, mobile device and health characteristics, and the association of the health and digital literacy of the participants.

Characteristics		Participants (n=81), n (%)	Digital literacy score (n=79), mean (SD)	*P* value^a^		Health literacy score (n=80), mean (SD)		*P* value^b^	
**Sex**					.28				.69
	Women	54 (67)	16.3 (6.7)			31.5 (7.7)			
	Men	27 (33)	18.6 (8.5)			30.9 (5.2)			
**Age group (years)**					.07				.15
	55-64	14 (17)	13.6 (4.5)			28.1 (7.3)			
	65-74	48 (59)	18.9 (8.2)			32.4 (6.5)			
	>75	19 (24)	14.9 (5.2)			30.7 (7.1)			
**Educational level (years)**					<.001				.01
	Primary education (1-4)	36 (44)	13.3 (5.2)			28.8 (5.9)			
	Basic education (5-9)	18 (22)	18.1 (8.0)			31.7 (8.2)			
	Secondary education (10-12)	12 (15)	19.3 (5.9)			33.7 (6.9)			
	Higher education (>12)	15 (19)	23.0 (7.4)			34.9 (5.8)			
**Marital status**					.10				.17
	Single	3 (4)	19.5 (9.3)			38.3 (10.6)			
	Married	57 (70)	17.1 (7.4)			31.8 (6.5)			
	Divorced	11 (14)	19.5 (7.4)			30.5 (7.7)			
	Widowed	10 (12)	13.2 (5.9)			27.0 (5.5)			
**Household composition**					.54				.77
	Single occupancy	18 (22)	17.7 (7.3)			32.4 (7.3)			
	Married/cohabiting with no dependent children	34 (42)	16.2 (7.6)			30.3 (7.0)			
	Married/cohabiting with dependent children	23 (28)	18.1 (7.3)			32.3 (7.0)			
	Single parent family	3 (4)	14.8 (10.2)			28.0 (7.8)			
	Another multi-person household	3 (4)	17.7 (6.0)			30.7 (2.3)			
**Job status**					.56				.97
	Retired	74 (91)	17.3 (7.4)			31.3 (6.7)			
	Employed	3 (4)	14.8 (8.5)			30.7 (4.1)			
	Unemployed	4 (5)	14.5 (6.1)			31.3 (12.6)			
**Mobile device purchaser^c^**					.009				.75
	Self	31 (48)	19.1 (7.7)			30.9 (6.6)			
	Other	33 (52)	14.5 (6.2)			30.4 (6.6)			
**Frequency of device use**					.02				.33
	Frequent	70 (86)	17.4 (7.2)			31.6 (6.9)			
	Infrequent	11 (14)	12.7 (8.6)			29.4 (7.8)			
**Type of mobile device used**					.004				.48
	Smartphone	72 (89)	17.8 (7.4)			31.5 (6.7)			
	Tablet	9 (11)	11.1 (2.4)			29.6 (8.8)			
**Mobile device operating system used**					.09				.25
	Android	71 (88)	16.5 (7.0)			31.6 (6.9)			
	iOS	10 (12)	21.1 (8.5)			28.9 (6.6)			
**Length of mobile device ownership**					<.001				.82
	More than 1 year	64 (79)	18.4 (7.3)			31.2 (6.7)			
	Less than 1 year	17 (21)	12.0 (5.2)			31.6 (8.0)			
**Regular physical exercise**					.16				.002
	Yes	58 (72)	17.6 (7.1)			32.7 (6.5)			
	No	23 (28)	15.7 (7.8)			27.7 (6.7)			
**Physical activity (per week)**					.34				.008
	>150 minutes	28 (35)	17.9 (7.3)			32.7 (5.0)			
	<150 minutes	28 (35)	17.6 (7.2)			33.3 (7.6)			
	0 minutes	23 (29)	15.7 (7.8)			27.7 (6.7)			
**Hypertension**					.77				.29
	Yes	46 (57)	17.3 (7.6)			30.6 (6.5)			
	No	35 (43)	16.7 (7.1)			32.2 (7.4)			
**Diabetes**					.43				.14
	Yes	11 (14)	15.9 (8.1)			28.3 (6.5)			
	No	68 (86)	17.2 (7.3)			31.8 (6.9)			
**Chronic pain**					.19				.11
	Yes	36 (45)	16.0 (7.1)			32.4 (6.6)			
	No	44 (55)	17.8 (7.5)			29.9 (7.2)			
**Alcohol habit**					.002				.96
	Yes	4 (5)	31.6 (5.2)			31.0 (5.6)			
	No	75 (95)	16.1 (6.6)			31.2 (7.0)			

^a^Data were analyzed with the Mann-Whitney Test (for dichotomous variables) or Kruskal-Wallis test (for polytomous variables).

^b^Data were analyzed with the 2-tailed *t* test (for dichotomous variables) or 1-way ANOVA (for polytomous variables). For the analysis of sex, the Welch *t* test (for violations of the assumption of equal variances) was used.

^c^Variable with general minimal sample.

### Study Feasibility

The intervention had an 81% (97/120) recruitment rate, 53% (43/81) adherence, and 94% (67/71) satisfaction. Analyzing the indicators by municipality showed that Barcelos had the best recruitment rate (95%, 19/20), Guimarães the best adherence rate (83%, 5/6), and Paredes de Coura the highest satisfaction rate (98%, 49/50).

### Longitudinal Digital and Health Literacy Analysis

**Figure 1:**
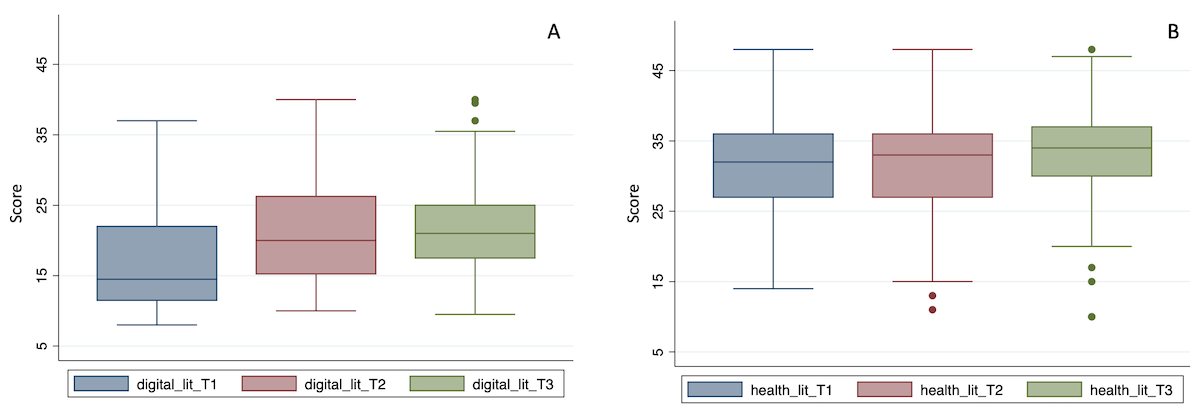
(A) Digital and (B) health literacy over time (T1, T2, and T3) among the participants, measured with the 16-question Mobile Device Proficiency Questionnaire and 12-question Health Literacy Scale instruments, respectively.

Analysis over time indicated a significant improvement in digital literacy, both immediately after the conclusion of the workshops (T2) and 1 month afterward (T3) compared to baseline (T1), but without significant differences in digital or health literacy scores between the postworkshop times (Figure 1). A significant improvement in self-reported autonomy was observed 1 month after the end of the workshops (T3) compared with baseline autonomy (T1), increasing from 4.5 to 6.7 points, with a score range from 0 to 10 (*t*_40_=–7.3; *P*<.001). Self-reported autonomy was evaluated with the following item on the questionnaire: “Circle the value corresponding to how autonomous you feel using a mobile phone with a touchscreen/tablet, with one being very little autonomous and ten being completely autonomous”; the response options were numbers with a color gradient from red to green. The health literacy analysis revealed no significant differences after the workshops compared to baseline.

The crude and adjusted analyses showed a significant association between digital literacy and health literacy at all study time points except the preworkshop adjusted analysis (Table 2).

**Figure 2:**
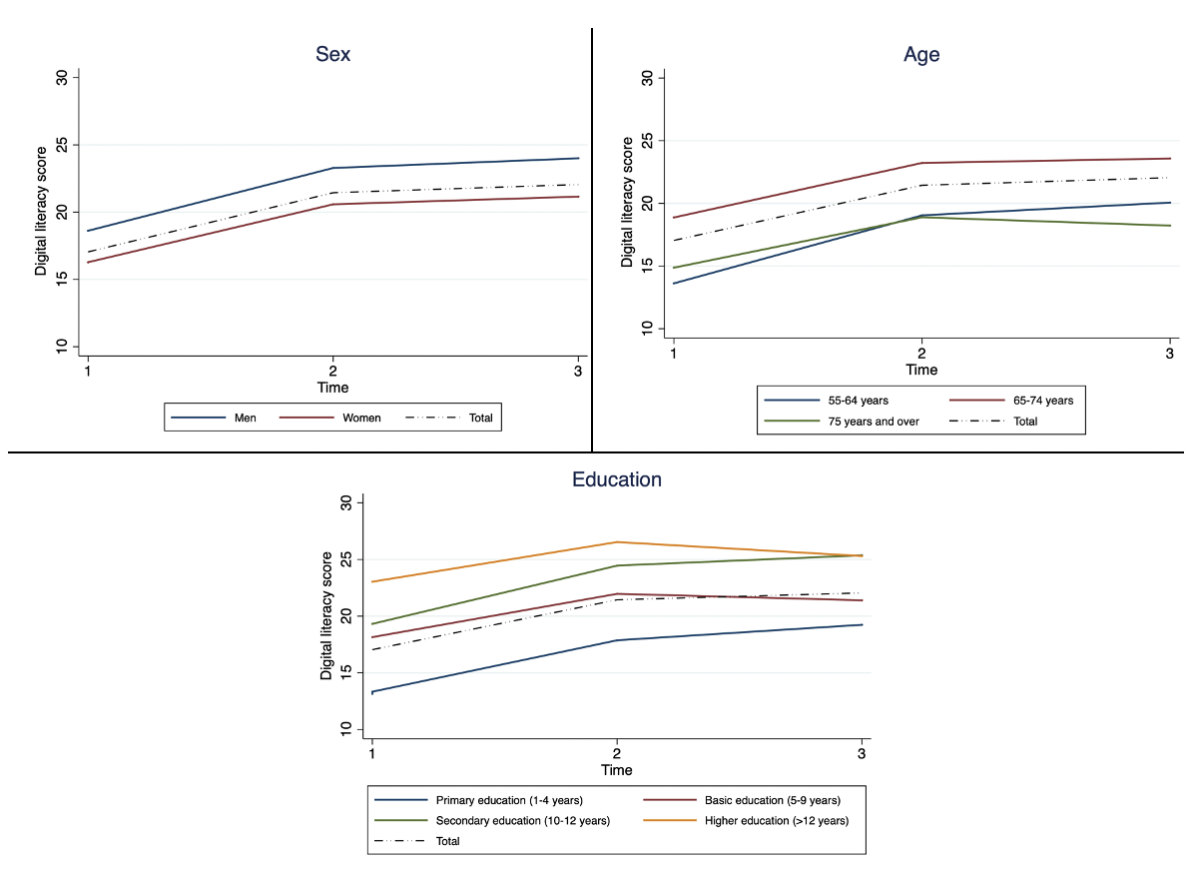
Digital literacy over time (at T1, T2, and T3) among the participants divided by sex, age, and education.

We performed a longitudinal analysis to explore digital literacy over time, adjusted by sex, age, and education level (Figure 2). Both women and men showed positive growth in both types of literacy. Moreover, all age categories showed improved postintervention scores, and the overall change in the performance of the participants was similar for all education levels. Specifically, there was an average increase of 2.49 points in the digital literacy score in the raw analysis and an average increase of 2.46 points in the analysis adjusted for sex, age, and education level. None of these variables revealed a significant interaction with time, meaning that the different subgroups varied similarly across the 3 time points (Table 3).

**Table 2:** Association between digital and health literacy at the 3 time points of the project.

Time point	Crude coefficient (95% CI)	*P* value	Adjusted coefficient^a^ (95% CI)	*P* value
Preintervention (T1)	0.34 (0.11 to 0.57)	.005	0.15 (–0.65 to 0.36)	.17
Postintervention (T2)	0.57 (0.36 to 0.77)	<.001	0.47 (0.27 to 0.67)	<.001
One-month postintervention (T3)	0.39 (0.15 to 0.62)	.002	0.32 (0.09 to 0.55)	.008

^a^Adjusted for sex, age, and education level.

**Table 3:** Analysis of digital literacy (based on Mobile Device Proficiency Questionnaire score) over time among the participants.

Variable	Crude analysis^a^			Adjusted analysis^a,b^	
	Coefficient (95% CI)	*P* value	Coefficient (95% CI)		*P* value
Time	2.49 (1.80 to 3.18)	<.001	2.46 (1.81 to 3.10)		.001
Sex	N/A^c^	N/A	–4.8 (–7.62 to –2.01)		.001
Age	N/A	N/A	0.09 (–0.15 to 0.32)		.46
Education level	N/A	N/A	2.40 (1.61 to 3.18)		<.001

^a^Adjusted for sex, age, and education level.

^b^Coefficient calculated with a generalized estimating equation model.

^c^N/A: not applicable.

## Discussion

### Main Results

The study aim was to develop a strategy for digital inclusion based on workshops that measured the digital and health literacy of middle-aged and older adult participants at 3 time points.

There are few studies on digital literacy assessment with intervention projects for older adults [8]. Most studies involving digital technologies refer to social networks or health apps [30,33-37]. Those who use apps for health care report low digital proficiency among older adults; app use is dependent on family members or caregivers [38-40]. The use of apps with mental health exercises has been explored, showing good feasibility and acceptance by middle-aged and older adults [35]. A study using an interactive TV app in Portugal showed that this intervention was feasible and acceptable for older Portuguese adults [34].

Here, we used an approach based on 8 workshops; the project methodology proved viable, with recruitment rate and satisfaction both greater than 80%. Moreover, we observed a significant improvement in digital literacy as measured by the MDPQ-16. Studies of mobile technology (ie, apps) have found low digital literacy among older adults [5,35,36,41]. These studies show advantages and improved outcomes when mobile technology and digital inclusion are used for health monitoring and treatment [21,33,42]. Other studies cite barriers that hinder the use of technology and report the importance of encouraging the use of these devices in the digital age [7,15,36,43]. Here, we observed that the participants’ baseline scores for digital proficiency were low. Even after the intervention, the participants reached, on average, 55% of the maximum score, with participants with low education (up to 4 years) having the lowest digital proficiency scores. As part of the Global Education 2030 Agenda initiative, UNESCO included guidelines to promote an environment of digital inclusion for the adult population after identifying the gap between digital evolution and the illiterate or poorly educated adult population. One of the UNESCO research objectives is to ascertain which skills these individuals need to effectively use inclusive digital solutions, as well as determine critical characteristics of the overall environment that are necessary to implement more inclusive solutions [2].

We included an analysis of health literacy in this study because of past findings that an increase in digital literacy favors the use of digital resources, including mHealth resources [19,44]. A study on using online health resources with smart devices showed that although 72% of older adults did not find the device to be a barrier to cardiac rehabilitation, only 18.4% used it for a health-related purpose [36]. Here, we noted no significant difference in health literacy across the project’s time points, but we did observe an association between digital literacy and health literacy at all time points of the assessment in the crude analysis. Still, the loss of the association between these two types of literacy in the baseline assessment, influenced by education, stands out in the adjusted analysis. A study with middle-aged adults indicated that literacy was positively associated with obtaining information from health professionals and the internet [45]. Another study demonstrated that middle-aged adults were 2.75 times more inclined to access eHealth services than young adults (aged 18-24 years), with participants with primary education only or a high school education being 72% and 62%, respectively, less likely to access eHealth services than participants with tertiary education [46]. Health promotion initiatives involving the use of technologies have been promoted worldwide. The WHO developed the Mobile Health for Aging program, and “mAgeing” was designed to manage age-related declines in intrinsic capacity and functional ability through mobile messages [47]. However, studies on the barriers faced by older adults to using mobile devices in their health care show that it is necessary to identify, among other factors, the motivational barriers preventing mHealth use by older adults [7].

Most study participants were women with primary schooling (up to 4 years) aged between 65 to 74 years who were retired. For the most part, community intervention studies have generally included female participants with low education [30,41,48]. Nevertheless, a study of factors associated with internet use showed that higher cognitive performance, being male, and being between 60 and 80 years old were determining factors for beginning internet use among Swedish older adults [49]. Here, even though our project allowed the participation of adults older than 55 years, there was greater participation by older adults. Being retired, they were available to participate in the intervention during the daytime. Older adults may also potentially be willing to accept internet-based interventions because they favor methods for digital inclusion that are easily applied in the community [50]. The workshops were the first group activity for many participants after a period of isolation caused by the COVID-19 pandemic. Initiatives using technologies such as video calls have been implemented to prevent loneliness, depressive symptoms, and isolation among older adults [42,51]. A study in Portugal showed that loneliness was diminished for internet users compared to their peers who did not use the internet [52].

Most participants in that study used a mobile device given or gifted to them by family members or others. The devices were in use by the participants for more than one year. However, it was also observed that the devices had previously been used by the person who offered them. Almost 90% of the participants used Android smartphones in the workshops. A study of digital inclusion for older adults showed that most participants were smartphone users, indicating that smartphones have gradually become a necessity [41], with the Android operating system considered the most prevalent among the target population of participants [53]. Most participants had healthy habits: 71.6% (58/81) practiced regular physical activity, 98.8% (78/79) were nonsmokers, and 94.9% (75/79) did not drink alcoholic beverages outside main meals. Older adult participants in community interventions are generally independent in activities of daily living, raising the question of how to promote inclusiveness among middle-aged and older adults with health or other impairments and dependency.

### Limitations and Strengths

Positive cases of SARS-CoV-2 infection occurred; one group in Paredes de Coura and another in Guimarães missed the T2 and T3 evaluations. Many participants used old smartphones with an operating system that could not be updated, and some could not install all the apps prepared for the workshops. Notably, 6 participants purchased newer smartphones because they felt the need to upgrade to enjoy the content of the workshops.

The feasibility indicators were satisfactory, showing that the methodology of the workshops allowed substantial rates of recruitment and satisfaction. The participants showed significant improvement in digital literacy after the 8 workshops, with their scores being maintained 1 month after the intervention ended.

The limitations of the study included not having a control group to compare with the intervention group. Moreover, the sample was not representative of sex, age group, or education by region or country; thus, the findings are not generalizable. Nonetheless, despite not having a representative sample, the variation in digital literacy was significant after controlling for sex, age, and education.

### Conclusions

In the study population, digital inclusion workshops with a group methodology approach significantly improved digital literacy after 8 workshops, albeit with no significant changes in health literacy. However, women and participants with lower education had lower digital literacy values. The OITO project methodology is feasible for community-dwelling adults aged 55 years or older.

## Abbreviations

EFA: exploratory factorial analysis
GEE: generalized estimating equation
HLS-12: 12-question Health Literacy Scale
KMO: Kaiser-Meyer-Olkin
MDPQ-16: 16-question Mobile Device Proficiency Questionnaire
mHealth: mobile health
Obercom: Communication Observatory
OITO: Oficinas de Inclusão Tecnológica Online (Workshops for Online Technological Inclusion)
WHO: World Health Organization

## Data Availability

The data set was published and is available in Mendeley Data [54].
